# Intention to use personal health record system and its predictors among chronic patients enrolled at public hospitals in Bahir Dar city, northwest Ethiopia: using modified UTAUT2 model

**DOI:** 10.3389/fmed.2024.1421559

**Published:** 2024-09-04

**Authors:** Solomon Abuhay Abebe, Berhanu Fikadie Endehabtu, Agmasie Damtew Walle, Debela Tsegaye Hailu, Ayenew Tilahun Yeshaneh, Eshetie Andargie Dres, Mengestu Tesfaye Yimmam, Kassahun Dessie Gashu

**Affiliations:** ^1^Department of Health Informatics, College of Medicine and Health Science, Hawassa University, Hawassa, Ethiopia; ^2^Department of Health Informatics, Institute of Public Health, College of Medicine and Health Science, University of Gondar, Gondar, Ethiopia; ^3^Department of Health Informatics School of Public Health, Debre Berhan University, Debre Birhan, Ethiopia; ^4^Department of Health Informatics, School of Public Health, Institute of Health, Bule Hora University, Bule Hora, Ethiopia; ^5^Jimma University Medical Center, Jimma, Ethiopia; ^6^Department of Health Informatics, Debre Tabor Health Science College, Debre Tabor, Ethiopia

**Keywords:** intention to use, PHR, chronic disease, UTAUT2, Ethiopia

## Abstract

**Introduction:**

Chronic diseases are the leading causes of death in the world. In sub-Saharan Africa, it leads to more mortality than almost every other region in the world. Currently, digital health technology like personal health records plays a crucial role in managing patients with chronic diseases. In low-resource countries like Ethiopia, it is uncertain how many chronic patients intend to use PHRs and the accompanying circumstances. Hence, the aim of this study was to assess chronic patients’ intention to use PHRs and its predictors enrolled in public health hospitals in Bahir Dar city, northwest Ethiopia.

**Method:**

An institutional-based cross-sectional study was conducted among 924 respondents from April 5 to May 9, 2023, in Bahir-Dar city public hospitals. A stratified sampling technique followed by a systematic sampling technique was applied to select the study participants. An interviewer-administrated questionnaire was conducted using Kobo Collect. A UTAUT2 model was applied to develop theoretical frameworks. SPSS version 25 software was used to estimate the descriptive statistics, and the structural equation model analysis was used to evaluate model constructs using AMOS version 21 software.

**Results:**

In this study, a total of 908 study subjects participated. The proportion of chronic patients’ intention to use PHR was 46.7% [95.0% CI (43.4–50.1)]. According to the findings, performance expectancy (*β* = 0.259, *p*-value <0.001), effort expectancy (*β* = 0.214, *p*-value <0.001), social influence (*β* = 0.174, *p*-value <0.001), and facilitating condition (*β* = 0.114, *p*-value <0.01) had a significant effect on the intention to use PHRs.

**Conclusion:**

Generally, the overall intention to use PHR was low. Our finding illustrates that the effects of performance expectancy, effort expectancy, social influence, and facilitating conditions had a positive effect on patients’ intentions to use PHRs. The effect of effort expectancy on the intention to use a PHR was positively moderated by age. Since the findings of this study would help policymakers and programmers to future academics interested in this area and insight to future research workers. Therefore, implementers should focus on improving patient capacity, motivating users, and raising awareness regarding PHR.

## Introduction

Chronic disease is an illness that develops over time and has persistent or long-lasting impacts when a condition has a longer than 3 months duration (1). It includes cardiovascular illness, chronic respiratory disease, hypertension, epilepsy, asthma, diabetes, etc. These diseases are a double burden in Africa, especially in sub-Saharan Africa, which has greater age-specific mortality rates from chronic diseases than almost every other region in the world (2). These nations bear a dual burden due to the growing issue of chronic disease; the total number of deaths from NCDs in sub-Saharan Africa is already larger than in many developed market economies. This role will put limited financial and human resources to the test, and if NCDs are neglected, their burden will only get worse. Using healthcare resources as cheaply and effectively as possible in all areas is more important than ever. In Ethiopia, with a higher share of morbidity and death, chronic diseases like cancer, diabetes mellitus, hypertension, pulmonary fibrosis, and cardiovascular problems are developing alarmingly quickly (3). In 2022, according to estimates from the World Health Organization (WHO), more than 43% of Ethiopia’s population will suffer from chronic diseases and/or pass away from them, with a countrywide prevalence of cardiovascular disease of around 15% (3, 4). The use of personal health records in the management of chronic diseases in Ethiopia may be complicated by the country’s limited access to technology and internet connectivity. It may be difficult for many people in Ethiopia to access and update their personal health records since they lack access to computers or smartphones, or their internet connectivity may be restricted. Inadequate or out-of-date health information may result from this lack of access, making it difficult for medical professionals to properly manage chronic illnesses. Moreover, problems with data security and privacy may make it more difficult to use personal health records in Ethiopia (5).

People with chronic conditions are expected to take a more active role in their health status (6). Patients have been urged to take more responsibility for their health and well-being by adopting eHealth tools as the prevalence of chronic diseases rises along with the development of information and communication technologies (7). Recent advancements have led to new breakthroughs in healthcare, including digital health, which is defined as the delivery of healthcare via mobile devices like personal health records (5). Management of chronic diseases continues to be one of the most significant issues facing the global healthcare industry today because of its effects on the individual, the community, and the economy. In response to the growing burden of chronic disease, clinical practice has placed a strong emphasis on the value of shared decision-making, active patient participation through self-management, and patient-practitioner interaction (8, 9). A person with a chronic disease engaging in activities that protect and promote health, monitoring and managing the symptoms and signs of illness, managing the impact of illness on functioning, emotions, and interpersonal relationships, and adhering to treatment regimens is the definition of self-management of a chronic disease. This strategy has decreased healthcare expenses while improving clinical outcomes and the effectiveness of health services.

As a result, the personal health record (PHR) is one of the most crucial self-care tools in this regard because it gives access to services for self-care as well as chronic disease management through observation and education (10).

A personal health record is a digital health management program that allows users to view, manage, and share their health information as well as the information of people they have permission to share within a private, secure, and confidential setting, including medical history, medications, medical prescriptions, and lab results (11). PHRs are a desirable and evolving technology that is becoming more and more popular across many nations in the context of health systems and applications (12). It is an electronic health record that allows users to securely, privately, and discretely access, manage, and share their own health information as well as the health information of those for which they are authorized (13). Furthermore, personal health records are created, owned, updated, and managed by an individual or an authorized person (13). A person’s lifelong medical history is summarized in their personal health record based on procedures, serious illnesses, allergies, blood pressure, data gathered from home monitoring devices, family history, vaccinations, prescription drugs, findings from laboratory testing, and other details (13). These applications, such as Microsoft health vault, patient like me, personal health monitor, HealtheVet, and my chart, are used for monitoring health conditions by tracking chronic information (10).

Chronic diseases are the leading cause of impairment and mortality worldwide, accounting for 75% of health care costs and 71% of global deaths (10). This is because many healthcare facilities maintain poor patient records that are stored in paper format and have an influence on both the patient’s and the doctor’s capacity to access the patient’s health information (14). In a resource-limited setting like Ethiopia, more than 43% of deaths are accounted for by non-communicable diseases (15). This was due to transportation inaccessibility, high expense, and lack of access to care, which are the challenges to visiting health facilities for follow-up (16). Currently, digital health technology like personal health records plays a crucial role in managing chronic patients (17).

The modern e-health system can be thought of as having several key components, one of which is healthcare information technology (HIT) (18). E-health includes several systems, such as personal health records, that deal with patient health information (17). As a result, some scholars suggest that personal health record technology is crucial for enhancing the quality of healthcare through the management of pressing global issues like chronic disease (17).

Despite the advancement of personal health record technologies, the study conducted in England showed that the overall adoption rate of PHR was low (19). The study conducted in America revealed that more than 55% of American customers who were not interested in utilizing PHRs said their hesitation was influenced by concerns about privacy and confidentiality (20). Another study conducted in Saudi Arabia revealed that several individual variables have also been identified as obstacles to implementing PHR devices. This was due to a lack of technical competency, chronic medical conditions, unrealistic expectations, and a lack of technology awareness (21–24).

Many factors, including rising medical expenses, advancements in medical technology, an aging population, and a rise in the prevalence of chronic diseases, have contributed to a shift in the way that health management is thought of (18). Due to this, patients have been urged to take more responsibility for their health and well-being by adopting eHealth tools as the prevalence of chronic diseases rises along with the development of information and communication technologies (11, 25). The goal of personal health records (PHRs) technology is to enable people to keep track of their health information and to improve patient involvement and empowerment (7). The study conducted in America showed that 79% of adult Americans believe that a PHR would significantly help patients manage their health. Another study conducted in America also showed that PHRs would also enhance cost-effectiveness; assuming 80% adoption of this technology in the United States, potential net savings are estimated at $19 billion annually. The study conducted in Europe showed that recent developments in personal health record (PHR) systems and health information technologies (HITs) have significantly changed how patients receive medical care. This has improved early disease detection, improved chronic disease management, and increased patient efficiency (26–28). According to the other study conducted in Portugal showed that by enabling people to take a more active role in their own care, personal health records (PHRs) seek to close the gap in the management of personal health information (29–31). And another study done in United Kingdom revealed that PHR systems can let patients’ message with doctors, make repeat prescription requests, and schedule appointments. In Estonia the study suggest that improved utilization of healthcare personnel and resources was one of the PHR system’s possible advantages (32).

In Africa, the research showed that patient, provider, and institutional benefits from personal health records are numerous. In addition, it enables patients to quickly access and download their medical records (33). The study done in Egypt showed that engagement through personal health records can aid in achieving the continuum of care and enabling healthcare service consumers to actively participate in the decision-making process and maintain health wellness and disease management (34).

Through economic reforms and initiatives to refocus health services to reach rural and impoverished populations in areas of health promotion, illness prevention, and curative care, the Ethiopian government has long sought to lessen economic disparities in the population. Among other things, these efforts have included new initiatives like the Health Extension Program (HEP) and health care finance techniques (35). There are now three tiers in the decentralized Ethiopian health system. These include a Woreda-based (district-based) system for primary care, which consists of a single primary hospital, health centers, and health posts. While care is provided in teaching and specialized hospitals at the tertiary level, general hospitals that are reached through referrals are included in the secondary care level (35).

The Ethiopian Ministry of Health (MOH) officially launched a Digital Health Innovation and Learning Center where specialists may develop and evaluate digital health solutions, compile and advance best practices, and scale up breakthroughs (36). However, in low-resource countries like Ethiopia, it is uncertain how many chronic patients intend to use personal health records and the accompanying circumstances. Therefore, this study aims to assess chronic patients’ intentions to use digital health technology, such as personal health records. Patients’ intention to use PHR and determining its predictors may be a crucial issue to facilitate healthcare delivery and a necessary condition to ensure the anticipated real utilization of health apps by chronic patients when we consider the introduction of PHR technology in Ethiopia (5).

Before introducing an emerging technology, it is important to assess the level of the intention of the potential users (37). According to our literature search, little is known about the intention of chronic patients in Bahir Dar city public hospitals to use PHR for self-care management. Therefore, this study would fill gaps according to chronic patients’ intentions to use PHR.

The findings of this study would help policymakers, programmers, and future academics interested in this area. It would also give insight to future researchers. On the other hand, this finding could also inform routine practices for healthcare providers and patients for chronic disease management using personal health records.

### Theoretical background and hypothesis

The Unified Theory of Acceptance and Use of Technology (UTAUT) is the model that has received the most attention to explain the relationship between independent and dependent variables. Even though there are other models available to explain user acceptance, Venkatesh et al. (38) created the UTAUT to offer a thorough framework to explain the acceptance and usage of information technology in organizations. It combines eight theoretical frameworks, including the Theory of Reasoned Action, the Technology Acceptance Model, the Motivational Model, the Combined Technology Acceptance Model-Theory of Planned Behavior, the Model of Personal Computer Utilization, the Diffusion of Innovation Theory, and the Social Cognitive Theory (38). To forecast the adoption and usage of technology, a unified theory of technology acceptance and use was presented in 2012 (39). As a result, selecting the appropriate theory or model as a theoretical basis to best explain user behavior toward the technology under investigation is critical to providing answers to the research questions. This study proposes a theoretical framework based on the UTAUT to examine the intention to use PHR due to its higher explanatory power.

Venkatesh et al. (38) evaluated the independent variables that influence behavioral intention and actual use of technology. Four constructs were proposed to impact user BI to utilize new technology: performance expectancy (PE), social influence (SI), effort expectancy (EE), and facilitating conditions (FC). Then, Venkatesh et al. (39) extended UTAUT and add three additional determinants, such as hedonic motivation (HM), price value (PV), and habit (HA), as well as individual variables including age, gender, and experience.

**Figure 1:**
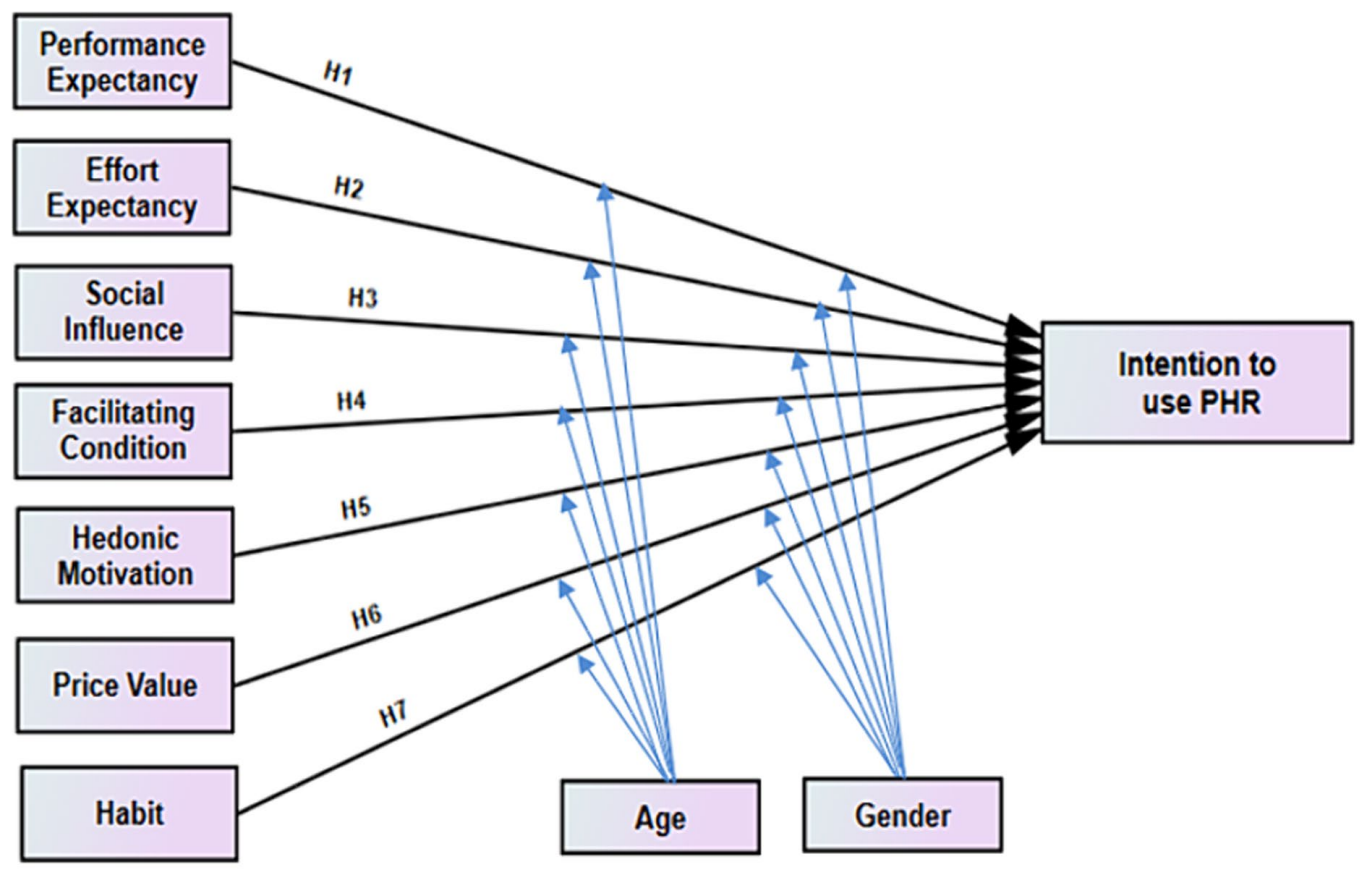
Conceptual framework for intention to use personal health record among chronic patients and its predictors enrolled at public hospitals in Bahir Dar city, Ethiopia, adapted UTAUT2 model.

We adopted the UTAUT2 model by including seven independent constructs and one dependent construct. Because the proposed technology is a predicted technology that has not yet been implemented in Ethiopia and because there are currently chronic patients who are not using PHR, user behavior, which was considered a dependent variable in the original UTAUT2, was not measured in this study (40). There are three parts to the conceptual research model. The first section contains UTAUT2’s seven exogenous variables: PE, EE, SI, FC, HM, PV, and HA. The second part of the model is made up of endogenous behavioral intention variables. Since the anticipated technology has not yet been fully implemented throughout Ethiopia and chronic patients have not yet fully utilized the PHR, this study did not measure user behavior, which was a dependent variable in the original UTAUT2 model (39). The third category consists of the moderators, which have an impact on both exogenous and endogenous factors like age and gender. Since the technologies are not implemented in Ethiopia, the people are not familiar with personal health records; thus, the experience was not included as a moderator. Finally, the proposed model is presented as follows (Figure 1).

The adapted constructs and the suggested hypothesis are discussed in more detail in the following subsections.

### Performance expectancy

The measure of how much a person believes that using technology will enable them to achieve major gains (7). A study conducted in England (41), Saudi Arabia (7), America (42) China (39, 43), Germany (44) and the USA (45, 46) performance expectancy was a positive predictor of behavioral intention on PHR adoption.

*H1*: PE has positively influenced chronic patients toward intention to use personal health records.

### Effort expectancy

The degree to which a person believes that using a particular technology will not necessitate a significant amount of effort (39). In a study conducted in Saudi Arabia (7), America (42), China (39, 43), Germany (44) and England (41), the basic structure of UTAUT is a significant predictor of the behavioral intention of the technology. The study done in the USA showed that a person’s intention to continue using a health and fitness app was not significantly impacted by the effort expectancy construct (45).

*H2*: EE has positively influenced chronic patients towards intention to use PHR.

### Social influence

It is the use of technology to assess a person’s perception of the significance of those in their social circle (47). It explains the successful persuasion of someone to utilize a particular technology by others (family, friends, or coworkers) (17). In a study conducted in Saudi Arabia (7), USA (46), China; Hong Kong’s social influence positively affected behavioral intention to use the technology (39, 43). However, in the study done in USA social influence have not significant to the behavioral intention to use health and fitness apps (45).

*H3*: SI has positively influenced chronic patients toward the intention to use PHR.

### Facilitating condition

The extent to which a person believes significant people believe they should use a particular technology (39). Which describes the facilities that might support a person’s use of the new technology (17). The study was conducted in China; Hong Kong’s facilitating conditions have a positive impact on behavioral intention to use technology (39). However, the study done in the USA on facilitating conditions has not been significant to patients’ intentions to use health and fitness technologies (45).

*H4*: FC has positively influence chronic patients toward intention to use PHR.

### Hedonic motivation

When a technological device is used, enjoyment and pleasure follow (39). The study conducted in the USA indicated that hedonic motivation was discovered to significantly affect behavioral intention in a recent eHealth study using UTAUT2 (45).

*H5*: HM has positively influenced chronic patients toward the intention to use PHR.

### Price value

Price value describes how price affects the decision to use new technology; individuals typically consider the price-to-performance ratio while making this decision (17). The study conducted in China showed that price value is positively associated with intending to use health information technology (39). In another study conducted in the USA price value was positively impacted by behavioral intention to use technology (45).

*H6*: PV has positively influenced chronic patients toward the intention to use PHR.

### Habit

Habit describes the user’s habitual behavior when using new technology; a person’s prior experience with similar technologies will encourage them to use the new technology (17). The study conducted in Malaysia indicated that habit is one of the most significant variables in determining how technology will be used in the near future. UTAUT2 has been widely used since its launch in 2012, with the exception of a glaring absence in the health sector (48). Also, the study conducted in the USA indicated that habit was a positive effect on the behavioral intention to use the technology (45). Another study done in China showed that UTAUT2, modeled habit as having an indirect effect through behavioral intention (39).

*H7*: Habit has positively influenced chronic patients toward intention to use PHR.

#### The moderating effect of age

The study conducted in Saudi Arabia indicated that all relationships were constrained by gender and age. It is anticipated that women and younger people have a stronger behavioral intention to use the PHR (7). Another study conducted in China showed that the moderator analysis demonstrated that there are distinct moderating effects of age groups on effort expectancy to behavioral intention to utilize technology (41). The study conducted in the U.S. state Age of the individual influenced the effects of PE, EE, and SI on behavioral intention to utilize health information technology (39). Another study conducted in America stated that another element that could affect a PHR system’s usability is age, and younger patients who regularly use computers are more likely to find new systems simple to use (49). The study conducted in England showed that Age moderates the positive link between Facilitating condition (FC) and user behavior (UB), making older females more susceptible to its influence (41). Another study conducted in the kingdom of Saudi Arabia indicated that age is not moderating effect on the intention to use PHR (7). Based on the finding the following hypothesis was proposed.

*H8*: The influence of PE on chronic patients’ behavioral intention to use personal health records has been moderated by age.

*H9*: The influence of EE on chronic patients toward intention to use PHR has moderated by age.

*H10*: The influence of SI on chronic patients toward intention to use PHR has moderated by age.

*H11*: The influence of FC on chronic patients toward intention to use PHR has moderated by age.

*H12*: The influence of HM on chronic patients toward intention to use PHR has moderated by age.

*H13*: The influence of PV on chronic patients toward intention to use PHR has been moderated by age.

*H14*: The influence of habit on chronic patients toward intention to use PHR has been moderated by age.

#### Moderating effect of gender

The study conducted in Florida, America, showed that gender is the only demographic characteristic that significantly associates with intending to adopt a PHR (50). Another study conducted in China indicated that sex had moderating effects on EE on behavioral intention to use the health system (51). Another study conducted in England indicated that the positive association between Effort Expectancy (EE) and patients’ intention to use PHR is moderated by gender (41). The study conducted in Saudi Arabia showed that the moderator’s gender does not affect the intention to use PHR (7). Another study conducted in China; Hong Kong showed that as the emphasis on instrumentality and gender variations in task orientation grow more evident (39). Therefore, the following hypothesis was proposed.

*H15*: The influence of PE on chronic patients’ behavioral intention to use personal health records has been moderated by gender.

*H16*: The influence of EE on chronic patients toward intention to use PHR has been moderated by gender.

*H17*: The influence of SI on chronic patients toward intention to use PHR has been moderated by gender.

*H18*: The influence of FC on chronic patients toward intention to use PHR has been moderated by gender.

*H19*: The influence of HM on chronic patients toward intention to use PHR has been moderated by gender.

*H20*: The influence of PV on chronic patients toward intention to use PHR has moderated by gender.

*H21*: The influence of habit on chronic patients toward intention to use PHR has been moderated by gender.

## Methods

### Study design and setting

An institutional-based cross-sectional study design was conducted from April 05 to May 09 2023. The study was conducted in Bahir Dar city’s public hospitals. Bahir Dar is the capital city of the Amhara regional state, which is located 570 kilometers northwest of Addis Ababa (52). The city is a popular tourist attraction in the nation and has a population of 280,780 people. By 2040, it is anticipated that Bahir Dar’s population would have multiplied more than four times (53). In addition to this, the city has three public hospitals: one primary hospital (Addis Alem), one comprehensive specialized referral hospital (Felege Hiwot), and one teaching hospital (Tibebe Ghion). There was a total of 6,500 follow-up chronic patients (hypertensive, diabetes mellitus, cardiac heart failure, and epilepsy) at the time the data were collected.

### Study participants and sample size determination

All adult chronic patients with diabetes mellitus, hypertension, epilepsy, and cardiac heart failure that had follow-up in Bahir Dar city public hospitals were used as a source population. Whereas all adult chronic patients with diabetes mellitus, hypertension, epilepsy, and cardiac heart failure that had follow-up in Bahir-Dar city public hospitals and were available during the study period were used as a study population.

The number of free parameters in the hypothetical model determines the minimum sample size; a 1:10 ratios of respondents to free parameters to be estimated has been recommended. Accordingly, considering the 84 parameters to be estimated based on the hypothesized model such as 36 variance of the independent variable, 21 covariance between independent variables, 20 load factors between latent-to-latent indicators, and 7 direct effects of regression coefficients between unobserved latent variables, and taking participants to a free parameter ratio of 10, the minimum sample required is 840. The sample size calculated accounts for the non-response rate of 10% and is therefore considered to demonstrate the final sample size. Thus, the final sample size becomes 924.

### Sampling procedure

The study participants were recruited from Bahir Dar city public hospitals. This study includes chronic non-communicable diseases that are more prevalent in terms of severity and death as well as more common in the three study hospitals based on reviewing the 6 months’ report, such as diabetes mellitus, hypertension, epilepsy, and cardiovascular disorders. The study participants were selected using stratified sampling technique followed by systematic sampling technique where, stratified sampling was done based on the type of disease. We used stratified sampling for the purpose of assign the sample allocation.

Proportional allocation done for public hospitals and participants were selected using a systematic random sampling method. The first patient was randomly selected using a lottery method followed by a selection of every study participant with the interval. Accordingly, every 7th patient was included in the study of the total adult (18 and above years) selected chronic disease patients. The final sample was selected from each stratum of a hospital using the proportional sample size allocation formula: Ni*n/*N* where: *Ni* = number of patients in each hospital, *n* = final sample of the study, *N* = the total number of patients in hospitals.

### Data collection tools

In this study of a PHR using UTAUT2, with little modifications to existing items to fit the objectives of the study. Responses to each question were provided on a 5-point Likert scale from 1 (strongly disagree) to 5 (strongly agree) (7). There are three sections to the questionnaire. Section A focuses on the socio-demographic characteristics with nine items each, Section B has five items on health-related aspects, and Section C has 28 positive statements that represent the components found in the UTAUT2 model. The 42 total questionnaires were measured using a five-point Likert scale, where 1 represents strongly disagree and 5 denotes strongly agree.

A well-structured questionnaire that was initially developed in English was developed by Kobo Collect software. As it is the intention of chronic patients to use PHR in Bahir Dar city, Amharic is the mother language, so the questions were translated into Amharic. The questions were translated from English into Amharic using the back translation technique in order to maintain the same originality and meaning in both languages. This step was important to ensure that the participants understood the survey questions and were not excluded because of language barriers.

### Data collection procedures

Data were collected using an interviewer-administrated technique. Three data collectors and one supervisor participated in the data collection process. A structured Amharic version of questionnaires was utilized by Kobo Collect software to collect data on chronic patients. Before the survey, trained investigators explained to the respondents about personal health records to help them understand the significance of the survey questions, and they either agreed or refused to take part in the study. Any respondent who did not give their oral consent was thanked for their time. Finally, the data collectors conducted interviews with those who had given their consent.

### Data quality control

Before the data collection date, data collectors and supervisors were given 2 days of training about the study’s purpose, data collection techniques, data collection tools, respondent approach, data confidentiality, and respondent rights using Kobo Collect software. The supervisors were examining the completeness and accuracy of the surveys every day. Before analyzing the data, it was cleaned up and cross-checked. Even though a questionnaire is a standard tool, it was pretested among a total of 10% of the total sample size of chronic patients at university of Gondar comprehensive and specialized hospital before data collection, and the result of reliability of latent variables was found to be above the Cronbach’s alpha and composite reliability threshold (0.7). Based on the results of the pretest little modification was made and the actual data collection was started.

### Data processing and analysis

The data was manually cleaned and coded by SPSS version 25 software before being sent for analysis. The Statistical Package for Social Science (SPSS) version 25 software was used to estimate the descriptive statistics of socio-demographic variables, health-related variables, and intention to use PHR. The structural equation model (SEM) analysis was used to evaluate model constructs using the analysis of moment structure (AMOS) version 21 software.

The sample size to be considered in SEM is large. The minimum sample size that should be utilized in the SEM method is at least 10 times the number of parameters that can be estimated in the model (54). In the SEM, it is assumed that there is no relationship between the independent variables, as the correlation between exogenous constructs should be less than 0.8. We used maximum likelihood to estimate the measurement model as well as the structural model. The measurement model was put to the test using confirmatory factor analysis (CFA) with standardized values, which illustrates how measured variables are combined to form constructs. Confirmatory factor analysis was used to verify the relationship between constructs and factor loadings for each item; the factor loading value for each item should be greater than 0.5 (5). Normality of data was assessed by applying multivariate kurtosis >5 and the critical ratio between −1.96 and +1.96 (55), but if this assumption is violated, it is recommended to use estimation methods such as bootstrapping methods (56). Assuming a normal distribution was utilized in this instance, the nonparametric test of bootstrapping methods aids non-normal data by resampling the data and estimating the significance of the path coefficients, standard errors, and confidence intervals. Thus, 5,000 bootstrap samples were used in AMOS, with a 95% bias-corrected confidence interval (57). And multicollinearity were also assessed by examining correlation and variance inflation factor (VIF) between variables. A VIF above 10 is indicator of multicollinearity whereas there was not a single VIF larger than 10, which suggests a lack of multicollinearity. VIF less than 10 and tolerance >0.1 as well as the correlation between exogenous construct less than 0.8 was employed (58). Additionally, the data were tested for normality using the Kolmogorov–Smirnov and Shapiro–Wilk test, which revealed that they were normally distributed when *p*-value is non-statistically significant (*p* > 0.05) else it is non normal distribution (7).

The parameter estimates were obtained using the maximum likelihood (ML) technique of approximation when the study’s variable showed normal behavior. However, it is advised to employ estimating techniques such as bootstrapping approaches if this assumption is violated (56).

In order to carry out the analysis effectively, the Kaiser–Meyer–Olkin (KMO) measure of sampling adequacy was checked to see if the sample items were appropriate for factor analysis. According to Kaiser Mayer Olkin, values between 0.5 and 0.7 are regarded as acceptable, 0.7 and 0.8 are good, 0.8 and 0.9 are excellent, and 0.9 and above are superb (KMO) (59). According to the finding of this study KMO value = 0.89.

Multivariate outliers were checked with the squared value of Mahala-Nobis distance (*d*^2^), and observations with a *p*-value less than 0.001 can be considered as multivariate outliers. Accordingly, outlier values were cross-checked in this study.

The goodness of fit of the models was evaluated using the chi-square ratio (≤5), normal fit index (NFI >0.9), comparative fit index (CFI >0.9), the goodness of fit index (GFI >0.9), adjusted goodness of fit index (AGFI >0.8), root mean square error approximation (RMSEA <0.08), and standardized root mean square of residual (SRMSR <0.08) (60).

The model was re-specified if the model fit indices were poor, and the model was improved by using a high value of modification indices to make the correlation between error terms until the model was fitted with a threshold value and no more than four times residual covariance allowed (61).

To assess the degree to which a variable is consistent and to evaluate how effectively the selected construct item measures the construct, reliability and validity were assessed. Construct reliability has been evaluated using the Cronbach alpha test, with each construct threshold of 0.70 and above, composite reliability exceeding 0.70, average variance extracted (AVE ≥0.5), and the factor loading value of each construct ≥0.5 (5). The average variance extracted (AVE) method was used to determine discriminant validity and values over the 0.50 cutoff, and the Fornell–Larcker criterion was used to assess discriminant validity. It was supported if a construct’s square root of AVE gives each corresponding bold value, and the bold values must be greater than each corresponding construct value row and column (5).

The link between exogenous and endogenous variables would be measured using squared multiple correlations (*R*^2^), and the standardized path coefficient, along with 95% confidence intervals and a *p*-value less than 0.05 to establish statistical significance.

The moderator, which can be continuous or categorical and is assessed through interaction effects and multiple group analysis, modifies the degree and direction of the link between the exogenous and endogenous variables (40). Multiple group analysis was used to evaluate the moderating effects of predictors among the hypothesized paths within the main research model because gender and age, were considered categorical variables in this study. Calculating the *p*-value and chi-square difference between the moderator’s effect and the unconstrained and constrained models used to distinguish weather the moderator is supported or not. Because the moderator in this study was evaluated using binary variables for gender and age. Age was categorized into groups for those under 40 and those above 40, accordingly (5, 62, 63). Using multiple group analysis, the moderating effects of predictors among the hypothesized paths within the basic research model were examined.

## Results

### Socio-demographic characteristics

In this study, a total of 908 (98.27% response rate) chronic patients participated. The median age of the participants was 48 [interquartile range (IQR): 35–61] years. Majorities 58.4% (530/908) of the study participants were older than 44 years old. From the study participants, about 50.7% (460/908) were males. Majorities: 70% (636/908) of the study participants were urban residents. Regarding marital status, 60.8% (552/908) of chronic patients were married. Majorities: 54.4% (494/908) of participants were orthodox. More than one-third of the study participants, 34.1% (310/908), were in higher education, as shown in Table 1 in detailed.

**Table 1:** Sociodemographic characteristics of study participants for intention to use personal health record among chronic patients in Bahir Dar city public hospitals 2023.

Sociodemographic characteristics	Category	Frequency (*N*)	Percentage
Gender	Male Female	460 448	50.7 49.3
Age (in years)	18–24 25–34 35–44 >44	52 168 158 530	5.7 18.5 17.4 58.4
Educational status	Unable to read and write Able to read and write Primary education Secondary education Higher education	235 131 89 153 310	24.8 14.4 9.8 16.9 34.1
Marital status	Single Married Separated divorced Widowed	135 552 58 60 103	14.9 60.8 6.4 6.6 11.3
Religion	Orthodox Muslim Protestant Catholic	494 226 152 36	54.4 24.9 16.7 4.0
Place of residence	Urban Rural	636 272	70.0 30.0
Occupation	Housewife Government NGO Farmer Merchant Student Others*	174 218 70 138 161 60 87	19.2 24.0 7.7 15.2 17.7 6.6 9.6
Family income	<5,000 5,000–9,999 10,000–15,000 >15,000	478 341 72 17	52.8 37.6 7.9 1.9
Distance to the health facility	<15 min 15–30 min >30 min	387 332 189	42.6 36.6 20.8

Others* contain daily laborers and unemployed.

### Clinical characteristics of chronic patients

The result showed that about 34.5% (313/908) of the study participants had hypertension, followed by 31.8% (289/908) of the study participants having diabetes mellitus. Regarding the duration of disease, about 41.7% (379/908) of the study participants were diseased for 3–6 years, followed by about 35.7% (313/908) of the study participants who were diseased for less than 3 years (Table 2).

**Table 2:** Clinical characteristics of the study participants at public hospitals in Bahir Dar city, 2023.

Clinical variables	Category	Frequency (*N*)	Percentage (%)
Type of chronic disease	Hypertensive Diabetes miletus Cardiac heart failure Epilepsy	313 289 189 117	34.5 31.8 20.8 12.9
Duration of chronic disease	<3 year 3–6 year >6 year	327 379 205	35.7 41.7 22.6
Follow up period	<3 year 3–6 year >6 year	459 340 109	50.6 37.4 12.0
Medication regularly	Yes No	721 187	79.4 20.6
Comorbidity	Yes No	221 687	24.3 75.7

### Intention to use personal health records

**Figure 2:**
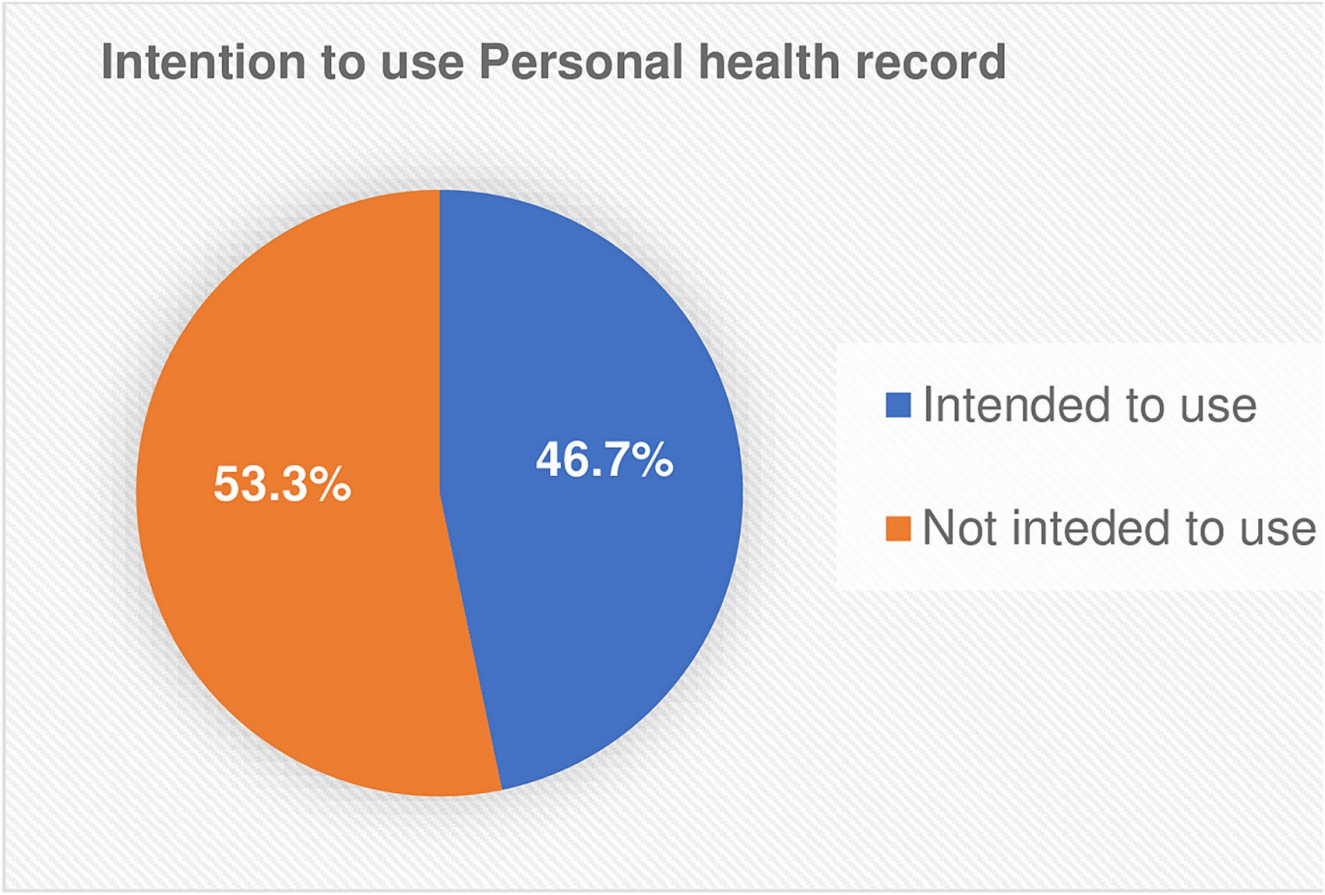
Proportions of intention to use personal health record among chronic patients in the public hospitals of Bahir Dar city, 2023.

In this study, the outcome variable (intention to use PHR) was measured by three questions with a five-point Likert scale. Intention to use personal health records was assessed by the median score of chronic patients who rated their intention to use PHR technology and scored median or above the median intended to use; otherwise, they are not intended to use. According to the findings, 424 (46.7%) (95.0%: CI: 43.4–50.0) of study participants, were intended to use personal health records. The median score of intention to use personal health records was 11 interquartile range (IQR): 6–12 and the maximum and minimum scores were 15 and 3 respectively, as shown in Figure 2.

### Measurement model assessment

**Figure 3:**
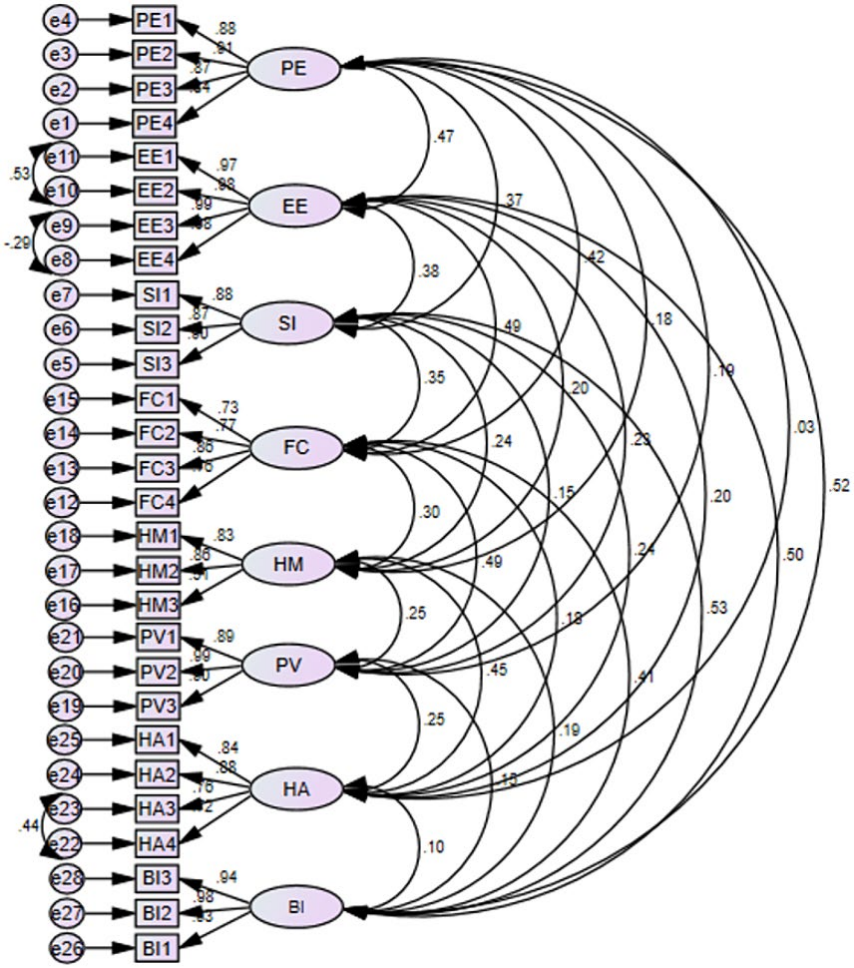
Confirmatory factor analysis (CFA) of intention to use PHR among chronic patients at public hospitals of Bahir Dar city, 2023.

Confirmatory factor analysis (CFA) is used to evaluate the measurement model by examining the indicators or items, such as model fit, internal consistency, convergent validity, and discriminant validity. To enhance model fit, we used covariate error terms with high modification indices. According to their highest modification indices, we draw covariances between error terms (e11 with e10, e9 with e8, and e23 with e22) (Figure 3).

In this study, the multivariate critical ratio does not range between −1.96 and +1.96 (CR = 53.4), and the multivariate kurtosis value is >5 (kurtosis = 102.1). Assuming a normal distribution was employed, the nonparametric test of bootstrapping methods here helps non-normal data by resampling the approach and assessing the significance of the route coefficients, standard errors, and confidence intervals (5, 57). Therefore, 5,000 bootstrap samples and 95% of bias-correlated confidence intervals were applied.

### Reliability and validity of the construct

The result shown in Table 3, the composite reliability ranges from 0.787 to 0.992 it shows that the suggested model’s construct reliability was attained. The table contains the Cronbach alpha from 0.726 to 0.992, indicating that the Cronbach alpha was satisfied with the models. Since the Cronbach alpha is one of the criteria to determine the internal consistency of the reliability. and the result finds the average variance extracted (AVE) values range from 0.563 to 0.970. On the other hand, the average loading of the 28 indicators or items varied from 0.51 for hedonic motivation to 0.99 for items related to price value and effort anticipation. Consequently, results demonstrated that convergent validity of the suggested model was attained. Fulfills the criteria of convergent validity it states that all the values of composite reliability and Cronbach alpha must be greater than 0.70 for all constructs. And also, average variance extracted (AVE) and factor loadings should be greater than or equal to 0.5. Therefore, the following table satisfies all the requirements of composite reliability, as shown in Table 3.

**Table 3:** Convergent validity between constructs for intention to use PHR among chronic patients in Bahir Dar city public hospitals, Ethiopia 2023.

	Indicators/items	Standard factor loading	Composite reliability (CR)	Cronbach alpha	Average variance extracted (AVE)
PE	PE1 PE2 PE3 PE4	0.88 0.91 0.87 0.84	0.930	0.929	0.768
SI	SI1 SI2 SI3	0.88 0.87 0.90	0.921	0.912	0.795
EE	EE1 EE2 EE3 EE4	0.97 0.98 0.99 0.98	0.992	0.992	0.970
FC	FC1 FC2 FC3 FC4	0.73 0.77 0.86 0.76	0.864	0.861	0.614
HM	HM1 HM2 HM3	0.83 0.86 0.51	0.787	0.726	0.563
PV	PV1 PV2 PV3	0.89 0.99 0.90	0.947	0.945	0.858
HA	HA1 HA2 HA3 HA4	0.84 0.88 0.76 0.72	0.878	0.890	0.643
BI	BI1 BI2 BI3	0.94 0.98 0.93	0.963	0.962	0.896

When assessing reflective conceptions, the Fornell–Larcker criterion is thought to be a more conservative approach than cross-loading analysis when determining discriminant validity. This criterion states that the squared correlation between any two constructs must be less than the square root of the AVE for each construct. Furthermore, an indicator’s outer loadings on a construct must exceed all of its cross-loadings with other constructs. For every construct in the model, the average variance extracted (AVE) which ranges from 0.563 to 0.970 was greater than 0.50. For every construct, the square root of the extracted average variance, which ranges from 0.783 to 0.985 (diagonal values), was likewise greater than its highest correlation with any other construct. Consequently, the discriminant validity of the model’s constructs was attained. Therefore, the result shown in Table 4 fulfills the Fornell–Larcker criterion (Table 4).

**Table 4:** Discriminant validity between constructs for intention to use PHR among chronic patients in Bahir Dar city public hospitals, Ethiopia 2023.

	AVE	PE	SI	EE	FC	HM	PV	HA	BI
PE	0.768	0.876							
SI	0.795	0.220	0.892						
EE	0.970	0.469	0.172	0.985					
FC	0.614	0.417	0.282	0.496	0.783				
HM	0.563	0.178	0.261	0.194	0.293	0.750			
PV	0.858	0.193	0.195	0.229	0.489	0.248	0.926		
HA	0.643	0.115	0.291	0.252	0.236	0.522	0.249	0.802	
BI	0.896	0.525	0.121	0.501	0.419	0.192	0.153	0.211	0.947

### Goodness of fit

Our model finds the model fit indices in confirmatory factor analysis of SEM model with comparing their threshold or respective values. If the result obtained reaches their threshold value, the model concluded that the model fit indices were excellent/ acceptable. According to the result our model obtained were chi-square per degree of freedom (CMIN/DF = 3.32), the goodness of fit index (GFI = 0.92), adjusted goodness of fit index (AGFI = 0.898), comparative fit index (CFI = 0.97), root mean square error approximation (RMSEA = 0.05), and standardized root mean square residuals (SRMSR = 0.043) Consequently, the values of the goodness of fit model satisfied the requirements (Table 5).

**Table 5:** Summary of model fit indices between constructs for intention to use PHR among chronic patients in Bahir Dar city public hospitals, Ethiopia 2023.

Fit indices	Threshold value	Results obtained	Conclusion
CMIN/DF	≤5	3.317	Acceptable
GFI	>0.9	0.920	Excellent
AGFI	>0.8	0.898	Excellent
CFI	>0.95	0.972	Excellent
RMSEA	<0.06	0.051	Excellent
SRMSR	<0.08	0.043	Excellent

### Structural equation model assessment

After determining the measurement model’s validity and ensuring there were no significant correlations between exogenous constructs, collinearity was verified before SEM analysis was utilized to evaluate the hypotheses. The variance inflation factor (VIF) and tolerance, which were used to test multicollinearity, Table 6 shows the multicollinearity test which indicates the tolerance value is greater than 0.1 and the VIF value is less than 10 (58). Therefore, the two value proofs there is no multicollinearity problem among independent variables (Table 6).

**Table 6:** Multi collinearity test between constructs for intention to use PHR among chronic patients in Bahir Dar city public hospitals, Ethiopia 2023.

Constructs	Tolerance	Variance inflation factor (VIF)
Performance expectancy (PE)	0.714	1.400
Effort expectancy (EE)	0.659	1.518
Social influence (SI)	0.780	1.282
Facilitating condition (FC)	0.613	1.630
Hedonic motivation (HM)	0.798	1.252
Price value (PV)	0.752	1.331
Habit (HA)	0.788	1.269

### Predictors associated with intention to use personal health record

**Figure 4:**
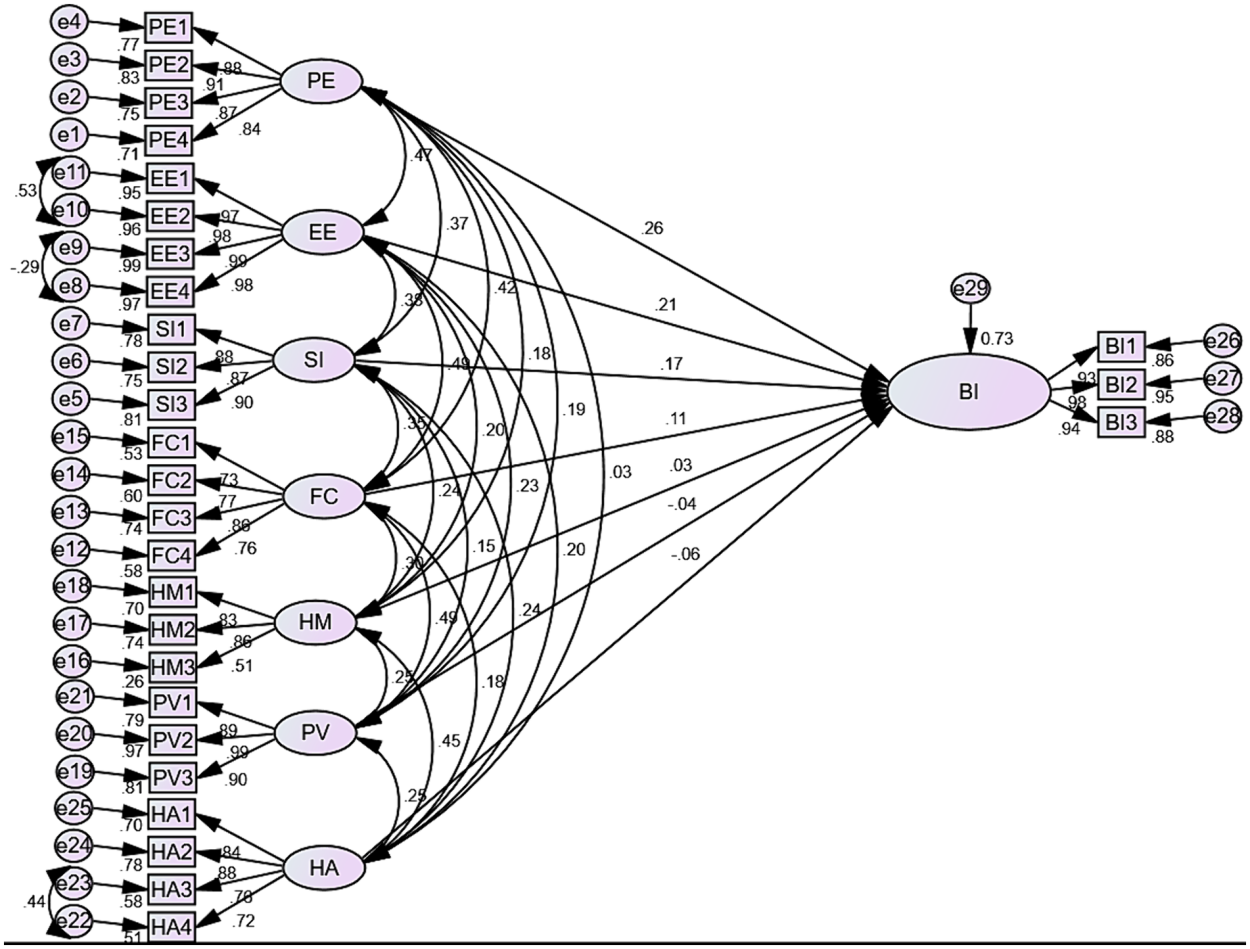
Predictors associated with intention to use PHR among chronic patients in Bahir Dar city public hospitals, Ethiopia 2023.

The figure below shows how to match the proportion of endogenous variables (intention to use personal health records) with the variance explained by exogenous variables such as performance expectancy, effort expectancy, social influence, facilitating condition, hedonic motivation, price value, and habit. Thus, the result shows the exogenous constructs explain 73% of the endogenous constructs (intention to use PHR). Therefore, the value of *R*^2^ is 73%. Figure 4 shows the standardized estimate of the model.

The result shown in Figure 4 shows that performance expectancy has the most substantial effect on the behavioral intention to use personal health records which had a larger effect than the other predictors. Having performance expectancy had a positive direct effect on the behavioral intention to use personal health records [*β* = 0.26, 95% CI: (0.150–0.540), *p*-value <0.001]. Thus, showing that an increase in chronic patients’ performance expectancy leads to an increase intention to use personal health records. Regarding effort expectancy, having effort expectancy had a positive direct effect on the behavioral intention to use personal health records [*β* = 0.21, 95% CI: (0.166–0.483), *p*-value <0.001]. Thus, finding leads to an increase in chronic patients’ effort expectancy, which leads to an increase in their intention to use personal health records. Similarly, social influence had a positive direct effect on the behavioral intention to use a personal health records [*β* = 0.17, 95% CI: (0.014–0.279), *p*-value <0.001]. Therefore, the result shows that an increase in the social influence of chronic patients leads to an increase in their intention to use personal health records. Regarding the facilitating condition, having a facilitating condition had a positive effect on the behavioral intention to use personal health records [*β* = 0.11, 95% CI: (0.055–0.234), *p*-value <0.01]; therefore, it indicates that an increase in the facilitating condition of chronic patients leads to an increase in the intention to use personal health records. In contrast, hedonic motivation [*β* = 0.03, 95% CI: (−0.076–0.167), *p*-value <0.381], price value [*β* = −0.04, 95% CI: (−0.053–0.203), *p*-value <0.166], habit [*β* = −0.06, 95% CI: (−0.101–0.197), *p*-value <0.092] had no direct effect on the intention of personal health records (Table 7).

**Table 7:** SEM analysis for predictors of intention to use personal health records among chronic patients in rolled at Bahir Dar city public hospitals, 2023.

Hypothesis	Estimate	Standard error (S.E.)	Critical ratio (C.R.)	*p*-value	95% confidence interval (CI)		Result
					Lower	Upper	
PE ➔ BI	0.259	0.038	5.936	0.00***	0.150	0.540	Accepted
EE ➔ BI	0.214	0.048	6.445	0.00***	0.166	0.483	Accepted
SI ➔ BI	0.174	0.035	5.846	0.00***	0.014	0.279	Accepted
FC ➔ BI	0.114	0.050	2.773	0.005**	0.055	0.234	Accepted
HM ➔ BI	0.032	0.051	0.075	0.381	−0.076	0.167	Not accepted
PV ➔ BI	−0.037	0.036	−1.386	0.166	−0.053	0.203	Not accepted
HA ➔ BI	−0.062	0.048	−1.683	0.092	−0.101	0.197	Not accepted

**p-value <0.01 and ***p-value <0.001.

### Testing potential moderators: gender and age

In this section, we investigate the moderators such as gender and age of chronic patients on the relation between performance expectancy, effort expectancy, social influence, facilitating condition, hedonic motivation, price value, and habit with the intention to use personal health records.

Multiple group analysis was done to identify the relationship between the exogenous constructs and endogenous variables by moderators’ gender and age. In multi-group analysis, there are two types of model comparisons: unconstraint and structural (constraint) model comparisons. The unconstraint model assumption showed that there is a moderator or statistical difference in the given variable to influence the endogenous and exogenous variables, whereas the structural weight (constraint) model suggests that there is no statistical difference between the endogenous and exogenous variables. If the *p*-value is less than 0.05 or the chi-square is >5, then the variable is confirmed as a moderator (5).

#### Moderating effect of gender

According to the result, there is no statistically significant difference between being male and female in terms of performance expectancy, effort expectancy, social influence, facilitating condition, hedonic motivation, price value, and habitual intention to use personal health records (Table 8).

**Table 8:** The moderating effect of gender for intention to use personal health records at Bahir Dar city public hospitals, 2023.

Hypothesis	Moderator	Path coefficient	*p*-value	Model test (constrained & unconstrained model)		Result
	Gender			Δ*X*^2^	*p*-value	
PE ➔ BI	Male Female	0.341 0.281	*** ***	0.755	0.385	Not accepted
EE ➔ BI	Male Female	0.215 0.232	*** ***	0.062	0.804	Not accepted
SI ➔ BI	Male Female	0.382 0.391	*** ***	0.018	0.893	Not accepted
FC ➔ BI	Male Female	0.181 0.126	*** 0.056	0.357	0.550	Not accepted
HM ➔ BI	Male Female	0.024 0.093	0.720 0.170	0.541	0.462	Not accepted
PV ➔ BI	Male Female	−0.100 −0.012	0.230 0.809	1.754	0.185	Not accepted
HA ➔ BI	Male Female	−0.160 −0.061	0.120 0.328	1.224	0.269	Not accepted

***Significant at *p*-value <0.001.

#### The moderating effect of age

According to the result below, the effect of effort expectancy on the intention to use personal health records was positively moderated by age and significantly at age ≤40 years (young age groups) of respondents (*β* = 0.222, *p*-value <0.001) compared to age group >40 years (old age groups) of respondents (*β* = 0.081, *p*-value <0.163). Whereas the effect of performance expectancy, social influence, facilitating condition, hedonic motivation, price value, and habit was not significantly different by moderator age (Table 9).

**Table 9:** The moderating effect of age for intention to use personal health record at Bahir Dar city public hospitals, 2023.

Hypothesis	Moderator	Path coefficient	*p*-value	Model test (constrained & unconstrained model)		Result
	Age			ΔX^2^	*p*-value	
PE ➔ BI	≤40 >40	0.310 0.297	*** ***	0.033	0.855	Not accepted
EE ➔ BI	≤40 >40	0.222 0.081	*** 0.163	6.74	0.047*	Accepted
SI ➔ BI	≤40 >40	0.345 0.369	*** ***	0.130	0.719	Not accepted
FC ➔ BI	≤40 >40	0.218 0.127	0.002** 0.028*	1.006	0.316	Not accepted
HM ➔ BI	≤40 >40	0.075 −0.009	0.264 0.888	0.834	0.364	Not accepted
PV ➔ BI	≤40 >40	−0.025 0.007	0.629 0.860	0.237	0.627	Not accepted
HA ➔ BI	≤40 >40	−0.151 −0.093	0.029 0.092	0.422	0.516	Not accepted

**Significant at *p*-value <0.01, ***significant at *p*-value <0.001, and *significant at *p*-value <0.05.

## Discussion

This study attempts to examine the intention to use personal health records and identify its predictors among chronic patients in the public hospitals of Bahir Dar city, north-west Ethiopia, using the UTAUT2 model. According to the result, about 424 (46.7) (95.0%: CI: 43.4–50.1) study participants intended to use personal health records. This result showed that less than half of chronic patients’ intention to use PHR to manage their health. The Ethiopian health sector digitalization program blueprint aims to prioritize and increase digitizing health information for patient and client benefits and also aims to establish the client’s use of health applications to manage their health and access to personal health records for the next 10 years (64). Even though the Ethiopian Ministry of Health established a digital health blueprint, our study’s patients’ intention to use their personal health records was still low. This might be due to the discrepancy in awareness of the use of personal health records. Another possible explanation for the gap is that nearly one-fourth (24.8%) of the respondents to this study were unable to read and write. In addition, Ethiopia has a low degree of emerging technology development, therefore, there are difficulties in understanding the use of medical apps, a lack of awareness regarding the significance of technologies, and a lack of e-health literacy (36).

Our suggested model explains 73% (*R*^2^ = 0.73) of the variance in chronic patients’ intentions to use personal health records. According to the finding, performance expectancy, effort expectancy, social influence, and facilitating conditions had a direct positive and significant effect on the intention to use personal health records. Accordingly, the hypotheses H1, H2, H3, and H4 are supported. Whereas hedonic motivation, price value, and habit did not significantly affect the intention of personal health records among chronic patients. The following findings describe, based on the results, how to enhance the intention to use personal health records in Bahir Dar city public hospitals.

According to the finding, performance expectancy had a direct positive effect on chronic patients’ intention to use personal health records (*β* = 0.259, *p*-value <0.001). This construct had a stronger relationship with the intention to use PHR than other constructs. This study provides credibility to the concept that patients find PHRs useful and are more likely to use them. This finding is in line with the previous studies done in England (41), Saudi Arabia (7, 13, 65), America (42), China (39, 43), and the USA (45, 46), German (44), Ethiopia (40). Additionally, it demonstrated a direct correlation between PHR systems and patient performance enhancements (40). This could be because people’s intentions to utilize PHRs at work are impacted by how beneficial they are for boosting everyday productivity and promoting patient safety (66). It might be PHR applications put the real health benefit front and center (44). Another reason might be due to the importance of personal health records supported by similar technologies. Another reason may be due to the serious prevalence of chronic diseases like hypertension, diabetes mellitus, cardiac-related diseases, and epilepsy, and they think personal health records are crucial to managing these diseases. Similarly, given that PHR technology is new to them, participants may expect that it will be perceived as useful (7). Patients will use PHR to improve healthcare services if they think it might be beneficial. By removing wait times, this technology improves the quality of healthcare. Additionally, users can maintain a greater rate of utilization and control of their health profiles through health profile management (13).

This study also showed that effort expectancy had a positive direct effect on chronic patients’ intentions to use personal health records (*β* = 0.214, *p*-value <0.001). This shows that when personal health records make it easier for participants to monitor their health and do healthcare tasks more rapidly, they are more likely to use them. This finding is consistent with other studies done in Saudi Arabia (7, 13), America (42), China (39, 43), and England (41), Germany (44), Ethiopia (40). This was due to people’s perception that users frequently choose technologies that are simple to use and accommodate their actual application requirements. It also be a result of their belief that employing PHRs will make their jobs easier and allow for the clear and methodical management of information and data (40). This might also be a result of participants believing they can use personal health records with little effort. This is also due to the fact that it has been demonstrated that a person’s intention to use technology is influenced by their expectation of how much work they will have to put into using a particular technology (39). Another point that may be put succinctly is people who find PHRs easy to use are more likely to use them frequently, which supports their impression of their importance and worth (13). However, this study opposes another study done in the United States (45). According to the literature ‘patients acceptance of health informatics applications’ differs of health professionals (65). This was due to patients’ have a low degree of self-efficacy and a poor opinion of the system’s usability as a result of the difficulties they have encountered when utilizing it (13). As a result, helping patients adopt PHRs is essential (13). The possible reason might be the improvement in the usability of technology, which lowers the potential effort required for utilization. Another significant predictor of intention to use personal health records is social influence (*β* = 0.174, *p*-value <0.001). This study is in line with the studies done in Saudi Arabia (25), China, and Hong Kong (37), Ethiopia (40). This might be because chronic patients believe that hospital administration, other patients, or medical staff may pressure them to use a personal health record (40). Another significant reason might be patients to become more motivated and intend to use PHRs in their company, they must feel pressure from an outside source. Patients’ acceptance of PHRs may be enhanced by peer support and role-modeling mechanisms like super users and champions (40). Therefore, patients with chronic conditions may feel pressure from an outside source to be more motivated and inclined to use personal health records. Patients’ intentions to use PHRs may rise if there are mechanisms in place that promote peer support and role modeling, such as supporters and powerful users (41).

Another finding of this study showed that facilitating conditions had a positive direct effect on the intention to use personal health records (*β* = 0.1141, *p*-value <0.01). The result showed that the availability of knowledge, resources, and support may motivate chronic patients’ intentions to use personal health records. This study was in line with the study done in China; Hong Kong’s facilitating conditions have a positive impact on behavioral intention to use technology (39). The potential cause could be that chronic patients think their medical professionals would be able to assist them by connecting with them so they can readily read and comprehend the output (39).

According to the finding, the relationship between effort expectancy and intention to use personal health records was positively moderated by age and significantly at age ≤40 years (young age groups) of respondents (*β* = 0.222, *p*-value <0.001) compared to age group >40 years (old age groups) of respondents (*β* = 0.081, *p*-value <0.163). For researchers, policymakers, and healthcare professionals, the finding that age modifies the relationship between effort expectancy and the intention to use Personal Health Records (PHRs) can have significant implications (5). This result showed that young age group respondents had a higher effort expectancy and intention to use personal health records than older age groups. This might be due to the fact that young age group participants had more interaction with technology. Another reason might be due to possible cognitive or technological hurdles, older persons may find PHRs more difficult to use, resulting in higher perceived effort expectancy. On the other hand, younger, more technologically literate people might think PHRs are simpler to use, which would lead to a lower perceived effort expectancy (67). Another possible reason might be that old age group patients are less likely to have exposure to developing technology. On the other hand, since they may have grown up with similar technology, younger chronic patients are more likely to feel more familiar with and understand the significance of personal health records (51). This finding was consistent with the research conducted in Saudi Arabia (7), China (41), U.S.A (39, 49).

### Practical implications of the study

In the real world, this study provides managers, producers, and healthcare sector decision-makers with useful information on how to increase chronic patients’ acceptance and usage of PHRs. PHRs and other eHealth technologies are being used in health care delivery with priority by the Ethiopian nation (68). Health care organizations across the nation will be asked to use PHRs to effectively provide person- and patient-centered care in order to accomplish the objectives of the National Transformation Program. Organizations may find ways to better manage patients’ health and well-being by using this study to better understand how patients view the PHR and develop patient engagement initiatives (7).

Additionally, it helps increase PHR adoption in order to lower the prevalence of chronic problems throughout Ethiopia. By making sure that healthcare services are completed more quickly and that upper management supports, allots resources, and imparts knowledge necessary for users to see the relative benefits of PHR technology, developers can improve the usability of personal health records in the healthcare industry. Lastly, this research provides PHR practitioners with a foundation upon which to develop healthcare policies that will promote the adoption of PHR medical technologies for self-care management.

Practitioners should focus on these predictors in order to actively develop a relationship with clients and encourage them to use the product on a regular basis, as performance expectancy, facilitating conditions, effort expectancy, and social influence all had a significant impact on a user’s intention to use personal health records. Health organizations might, for example, draw attention to the customized advantages and market features that may facilitate health management.

## Conclusion

The overall proportion of chronic patients’ intentions to use personal health records was low. Performance expectancy, effort expectancy, social influence, and the presence of a facilitating condition all served as statistically significant predictors of chronic patients’ intention to use a personal health record. Among the four influential predictors, performance expectancy had a greater predictive power of patients’ intentions to use personal health records. Age had a positive moderating effect on the relationship between effort expectancy and intention to use PHRs. The results of this study have important implications for personal health record program design, intervention planning, and research concerns for chronic patients. The finding of this study is important for policy makers, for Bahir Dar city public hospital managements, for future researchers, and for patients.

### Limitations of the study and future research

First, since the study was an institutional-based cross-sectional study, the result may not show a cause-and-effect relationship. Secondly, the study focuses only on Bahir Dar city public hospitals, therefore; the study findings may be limited by the specific sample representation, which only includes data from a particular geographical area or healthcare setting. This limitation suggests that the future studies should aim to include a broader geographical area or different type of health care settings like adding private hospitals and rural clinics to enhance the generalizability of the results. Finally, because the study was taken at a few institutions, it’s difficult to generalize.

## Data Availability

The raw data supporting the conclusions of this article will be made available by the authors, without undue reservation.
